# Exenatide reduces atrial fibrillation susceptibility by inhibiting hKv1.5 and hNav1.5 channels

**DOI:** 10.1016/j.jbc.2024.107294

**Published:** 2024-04-16

**Authors:** Qian Zhou, Guoliang Hao, Wensen Xie, Bin Chen, Wuguang Lu, Gongxin Wang, Rongling Zhong, Jiao Chen, Juan Ye, Jianping Shen, Peng Cao

**Affiliations:** 1 Jiangsu Provincial Medical Innovation Center, Affiliated Hospital of Integrated Traditional Chinese and Western Medicine, Nanjing University of Chinese Medicine, Nanjing, China; 2 Institute of Electrophysiology, Henan Academy of Innovations in Medical Science, Zhengzhou, China; 3 Henan SCOPE Research Institute of Electrophysiology Co Ltd, Kaifeng, China; 4 Burdon Sanderson Cardiac Science Centre and BHF Centre of Research Excellence, Department of Physiology, Anatomy and Genetics, University of Oxford, Oxford, UK; 5 Nanjing Research Institute for Comprehensive Utilization of Wild Plants, Nanjing, China; 6 State Key Laboratory on Technologies for Chinese Medicine Pharmaceutical Process Control and Intelligent Manufacture, Nanjing University of Chinese Medicine, Nanjing, China; 7 Animal-Derived Chinese Medicine and Functional Peptides International Collaboration Joint Laboratory, Nanjing University of Chinese Medicine, Nanjing, China; 8 Shandong Academy of Chinese Medicine, Jinan, China

**Keywords:** exenatide, hKv1.5, close channel block, hNav1.5, open channel block, atrial fibrillation

## Abstract

Exenatide, a promising cardioprotective agent, protects against cardiac structural remodeling and diastolic dysfunction. Combined blockade of sodium and potassium channels is valuable for managing atrial fibrillation (AF). Here, we explored whether exenatide displayed anti-AF effects by inhibiting human Kv1.5 and Nav1.5 channels. We used the whole-cell patch-clamp technique to investigate the effects of exenatide on hKv1.5 and hNav1.5 channels expressed in human embryonic kidney 293 cells and studied the effects of exenatide on action potential (AP) and other cardiac ionic currents in rat atrial myocytes. Additionally, an electrical mapping system was used to explore the effects of exenatide on electrical properties and AF activity in isolated rat hearts. Finally, a rat AF model, established using acetylcholine and calcium chloride, was employed to evaluate the anti-AF potential of exenatide in rats. Exenatide reversibly suppressed *I*_Kv1.5_ with IC_50_ of 3.08 μM, preferentially blocked the hKv1.5 channel in its closed state, and positively shifted the voltage-dependent activation curve. Exenatide also reversibly inhibited *I*_Nav1.5_ with IC_50_ of 3.30 μM, negatively shifted the voltage-dependent inactivation curve, and slowed its recovery from inactivation with significant use-dependency at 5 and 10 Hz. Furthermore, exenatide prolonged AP duration and suppressed the sustained K^+^ current (*I*_ss_) and transient outward K^+^ current (*I*_to_), but without inhibition of L-type Ca^2+^ current (*I*_Ca,L)_ in rat atrial myocytes. Exenatide prevented AF incidence and duration in rat hearts and rats. These findings demonstrate that exenatide inhibits *I*_Kv1.5_ and *I*_Nav1.5_*in vitro* and reduces AF susceptibility in isolated rat hearts and rats.

Atrial fibrillation (AF), a highly prevalent cardiac arrhythmia, is a major risk factor for ischemic stroke, imposing a substantial economic burden along with high morbidity and mortality. The global prevalence of AF has increased substantially over the past 3 decades, with approximately 60 million cases worldwide (1). Current AF management approaches include rate and rhythm control strategies as well as surgical interventions. Rate control strategies are useful in reducing the ventricular rate with the goal of alleviating related symptoms but without converting the heart to a regular rhythm. Rhythm control strategies seek to suppress ectopic activity, interrupt re-entry, and convert the heart to sinus rhythm by mainly using antiarrhythmic drugs. Antiarrhythmic drugs are important in the management of AF because of their noninvasiveness and low cost compared to ablation therapy (2). However, the currently available antiarrhythmic medications are not fully effective and are burdened with a major risk of cardiac and extra-cardiac adverse effects (3).

*SCN5A* encodes the cardiac sodium channel hNav1.5 which is responsible for the initiation and propagation of action potential and thus determines cardiac excitability and conduction throughout the atria and ventricles (4, 5). When atrial vulnerability is increased, atrial ectopic beats generate excitatory impulses that can result in reentrant circuits and AF initiation. Accordingly, sodium channel blockers decrease the occurrence of atrial ectopic beats and episodes in patients and experimental studies with AF (6, 7, 8).

Kv1.5, encoded by *KCNA5*, confers the cardiac ultra-rapid delayed-rectifier potassium channel current *I*_Kur_ and is specifically expressed in human atria, but not in ventricles, thereby contributing to a predominant effect on the action potential duration (APD) and effective refractory period (ERP) in the human atrium (9, 10). However, pharmaceutical investigations have not obtained direct evidence to show that sole Kv1.5 blockade is sufficient for suppressing AF in patients (11). Recent experimental and clinical studies have shown that a combined blockade of hNav1.5 and hKv1.5 channels on the heart is more effective than a specific blockade of just one target for managing AF (12, 13). For instance, the addition of an *I*_Kur_ blocker improved the atrium-selective electrophysiological profile and anti-AF effects of *I*_Na_ blockade in canine atrial tissue preparations (14). Simultaneous blockade of the *I*_Na_ and atrial-specific *I*_Kur_ had synergistic anti-AF effects, without inducing significant QT prolongation and ventricular adverse effects (15).

Glucagon-like peptide 1 (GLP-1), a gut-derived peptide hormone secreted in response to meal ingestion, exerts insulinotropic, glucagonostatic, and satiety-promoting effects as well as a delaying effect on gastric and intestinal motility (16). Exenatide is a synthetic GLP-1 receptor activator derived from exendin-4 isolated from the saliva of the Gila monster (*Heloderma suspectum*) that is used to treat symptoms and complications of diabetes mellitus (17). Exenatide at therapeutic and supratherapeutic concentrations does not prolong the corrected QT in healthy individuals (18). In addition, exenatide treatment can preserve cardiac function and attenuate structural remodeling in humans and rodents (19, 20). Moreover, exenatide protects cardiomyocytes against oxidative stress-induced injury (21). However, whether exenatide has an anti-AF effect and the underlying electrophysiological mechanisms remain unknown.

The present study was designed to investigate the effect of exenatide on AF *in vitro* and *in vivo*. First, we determined the potency of exenatide on hKv1.5 and hNav1.5 channels expressed in human embryonic kidney 293 (HEK 293) cells. Moreover, we investigated the effects of exenatide on action potential and other cardiac ionic currents in adult rat atrial myocytes. Additionally, we assessed the effect of exenatide on AF susceptibility in acetylcholine- and pacing-triggered isolated rat hearts and in rats treated with acetylcholine and calcium chloride (acetylcholine-CaCl_2_).

## Results

### Exenatide inhibits hKv1.5 current

**Figure 1:**
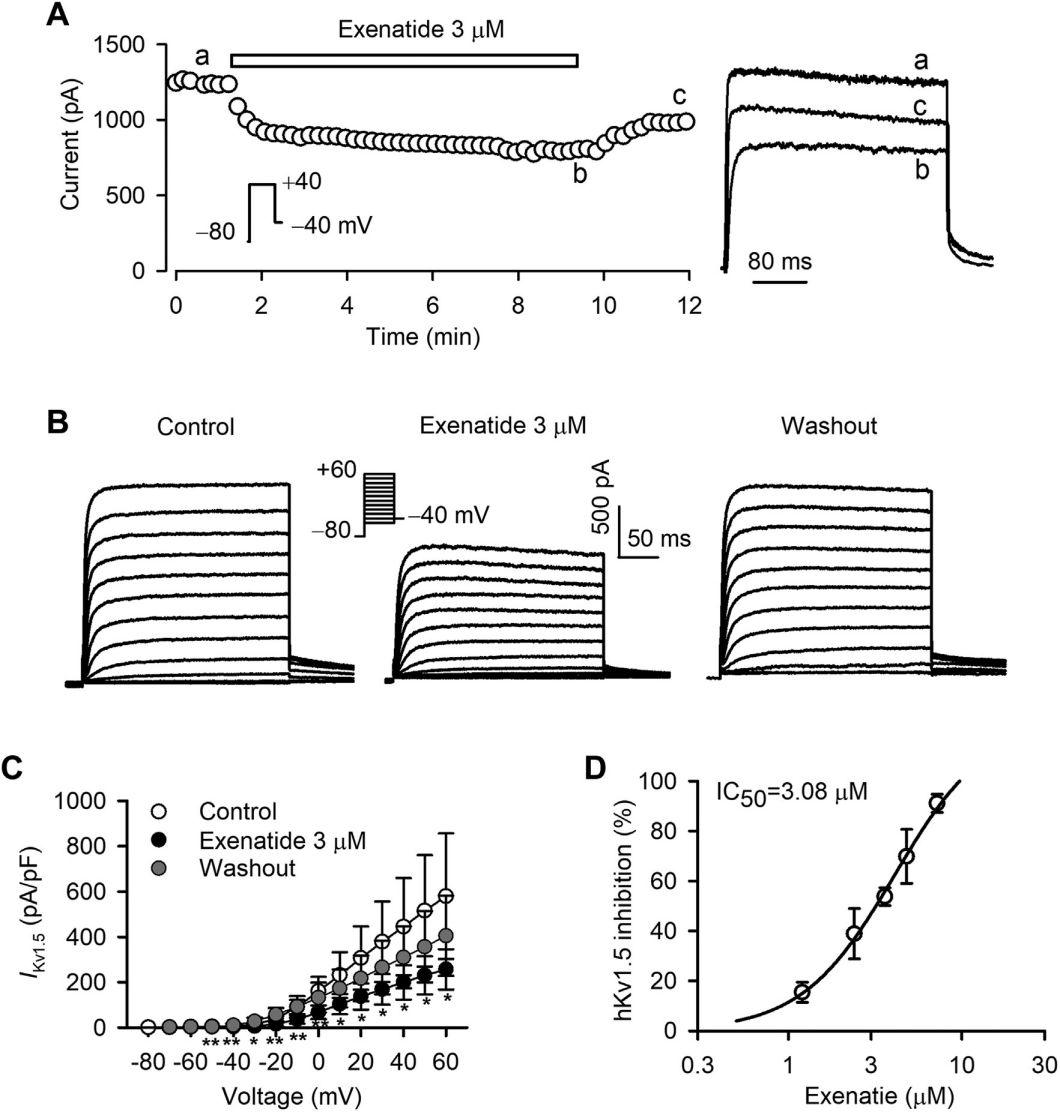
**Effect of exenatide on hKv1.5 current.***A*, time dependence of 3 μM exenatide on hKv1.5 current elicited by a 300-ms voltage step to +40 mV from −80 mV (left inset) delivered every 10 s in a typical experiment. Right inset: original current traces at the corresponding time points of (*A*). *B*, voltage-dependent hKv1.5 traces recorded in a representative cell with control, 3 μM exenatide, and after washout. *C*, current-voltage (*I-V*) relationships of hKv1.5 current with control, 3 μM exenatide, and after washout (n = 5; *p* < 0.05 or *p* < 0.01 *versus* control, paired Student’s *t* test). *D*, concentration-response curve of exenatide in inhibiting hKv1.5 current at +40 mV (n = 3–9). Symbols are the mean values of inhibitory effects in cells exposed to different concentrations of exenatide. Data points were fitted to the Hill equation.

Figure 1*A* shows the time course of hKv1.5 current recorded in a representative HEK 293 cell expressing KCNA5 in the absence or presence of 3 μM exenatide using a 300-ms voltage step to +40 mV from a holding potential of −80 mV. Exenatide gradually reduced hKv1.5 current, and this inhibition was rapidly reversed (80.62%) on washout. The right inset shows the original traces of the hKv1.5 currents at the corresponding time point of Figure 1*A*. Voltage-dependent hKv1.5 traces were recorded in a representative experiment with control (approximately 5 min for initial phase), 3 μM exenatide (approximately 7 min for stable effect), and after washout (approximately 3 min), using the voltage protocol shown in the inset (Fig. 1*B*). Both step and tail currents of hKv1.5 were substantially decreased by exenatide and the inhibitory effect was reversed upon washout. It should be noted that both the peak and steady state of hKv1.5 currents were concurrently inhibited by exenatide, suggesting that exenatide may be a closed channel blocker. Current*-*voltage (*I-V*) relationships of hKv1.5 current in the absence or presence of 3 μM exenatide are plotted in Figure 1*C*. The hKv1.5 current density was significantly inhibited by exenatide (n = 5; *p* < 0.05 or *p* < 0.01 *versus* control at −50 to +60 mV), and this effect could be washed out. Exenatide reversibly suppressed hKv1.5 current in a concentration-dependent manner with a half-maximal inhibitory concentration (IC_50_) of 3.08 μM and Hill coefficient of 2.2 (Fig. 1*D*).

### Blocking properties on hKv1.5 current by exenatide

**Figure 2:**
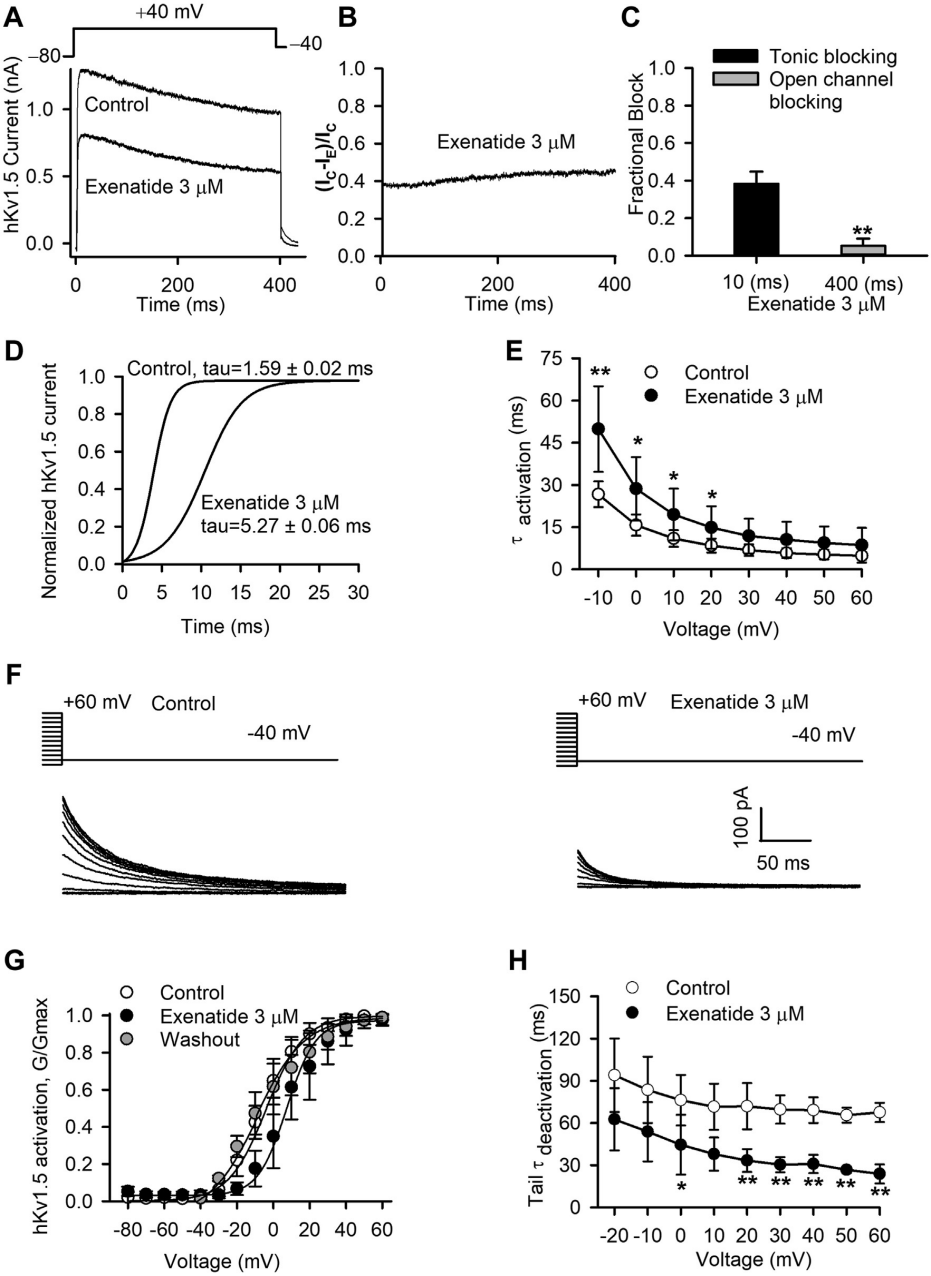
**Blocking properties of exenatide on hKv1.5 current.***A*, protocol and representative hKv1.5 current traces recorded with control and 3 μM exenatide. *B*, drug-sensitive current expressed as a proportion of the current in the absence (I_C_) and presence of 3 μM exenatide (I_E_). *C*, mean values of fractional block for the initial tonic blocking and open channel blocking with 3 μM exenatide (n = 5; *p* < 0.01, paired Student’s *t* test). *D*, normalized current of the expanded hKv1.5 activation phase before and after 3 μM exenatide treatment in a typical experiment. *E*, voltage dependence of activation time constants of hKv1.5 current before and after 3 μM exenatide (n = 5; *p* < 0.05 or *p* < 0.01 *versus* control, non-paired Student’s *t* test). *F*, protocol and tail current traces used to assess the conductance of hKv1.5 channels before and after 3 μM exenatide treatment. *G*, normalized hKv1.5 tail (G/Gmax) variables with control, 3 μM exenatide, and after washout fitted to the Boltzmann equation. *H*, mean values of voltage-dependent time constants of the deactivation tail decay of hKv1.5 channels before and after 3 μM exenatide (n = 5; *p* < 0.05 or *p* < 0.01 *versus* control, non-paired Student’s *t* test).

A 400-ms voltage step to +40 mV from a holding potential of −80 mV and then back to −40 mV, was applied to analyze the blocking property of exenatide, as previously described (22) (Fig. 2*A*). It was observed that exenatide decreased *I*_Kv1.5_ within a 10 ms activation, which is characteristic of a tonic blocker. To further analyze the onset of channel blockade, the drug-sensitive current formula (23) (*I*_C_-*I*_E_)/*I*_C_ was plotted against time, where *I*_C_ and *I*_E_ are currents in the absence and presence of exenatide, respectively. The results showed that the inhibition by exenatide observed within 10 ms (tonic blocking) was similar to that seen at 400 ms (Fig. 2*B*). A very small fraction of open channel blocking was obtained by subtracting the current at 10 ms from that at 400 ms (Fig. 2*C*; n = 5; *p* < 0.01). As the closed channel blocking effect is typically associated with slowed activation, we, therefore, analyzed the activation time constant of hKv1.5 current by fitting data to a monoexponential equation before and after 3 μM exenatide in a representative cell. Exenatide significantly delayed hKv1.5 channel activation, with the activation time constant increased from 1.59 ± 0.02 ms for control to 5.27 ± 0.06 ms for exenatide (Fig. 2*D*). A significant difference in activation time constant was observed at tested potentials from −10 to +20 mV (Fig. 2*E*; n = 5; *p* < 0.05 or *p* < 0.01 *versus* control).

Steady-state activation conductance (G/Gmax) of hKv1.5 was determined by analyzing the deactivation tail current in the absence and presence of 3 μM exenatide (Fig. 2*F*). The *V*_1/2_ of *I*_*K*v1.5_ activation positively shifted by 15.93 mV after the application of 3 μM exenatide (2.52 ± 2.39 mV for control vs. 18.45 ± 1.9 for exenatide; n = 5; *p* < 0.05) and negatively reversed to 7.24 ± 2.14 mV after washout (n = 5) (Fig. 2*G*). The slope factor value (κ) was not significantly altered by exenatide treatment. The voltage-dependent deactivation time constant of hKv1.5 was reduced by 3 μM exenatide with statistical significance at 0 to +60 mV (n = 5; *p* < 0.05 or *p* < 0.01 vs. control) (Fig. 2*H*), suggesting that exenatide facilitates the deactivation process of the hKv1.5 channel. However, use- and frequency-dependent inhibition of hKv1.5 current was not observed with 3 μM exenatide at 1, 2, and 4 Hz when compared to the control (Fig. S1; n = 4; *p* > 0.05), which supports the notion that exenatide inhibited the hKv1.5 channel in the closed state.

To exclude the possibility that patch duration would affect exenatide-induced inhibition of hKv1.5 channel, hKv1.5 current was recorded in cells treated with control after a stable current was reached (after approximately 5 min of membrane rupture, initial phase) until an additional 15 min (Fig. S2*A*). The hKv1.5 current was slightly reduced by 4.57 ± 3.29% (n = 3; Fig. S2*B*). The *V*_1/2_ of *I*_Kv1.5_ activation was negatively shifted from −4.86 ± 0.89 mV at the initial phase to −7.11 ± 1.37 mV at the additional 15 min (Fig. S2*C*; n = 6; *p* > 0.05). These results indicated that the potential influence of patch duration on exenatide-induced reduction of hKv1.5 current is limited.

### Exenatide blocks hNav1.5 current

**Figure 3:**
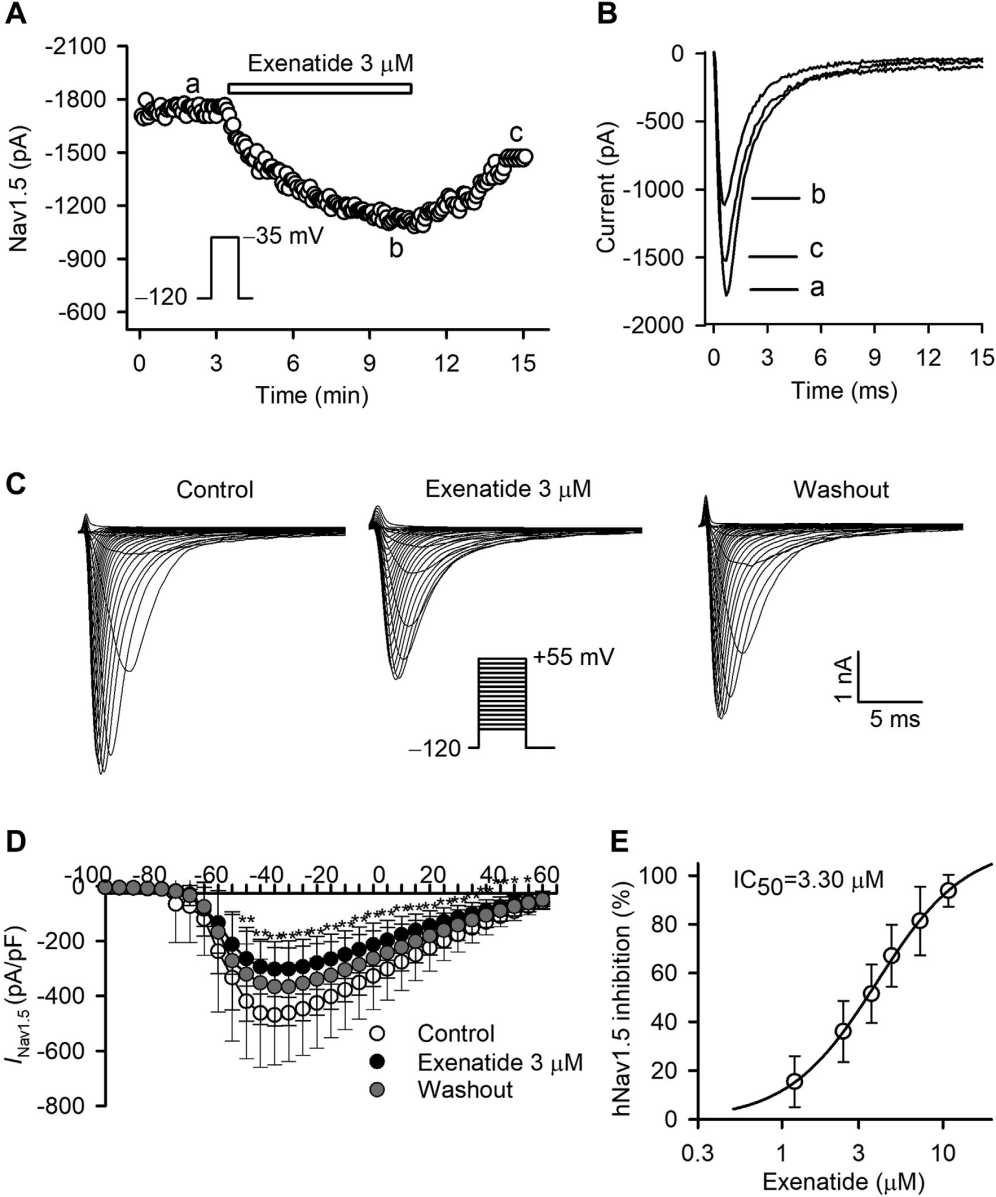
**Exenatide blocks hNav1.5 current.***A*, time course of hNav1.5 current with and without 3 μM exenatide using a 100-ms test pulse from −100 to −35 mV and back to −120 mV in a typical cell. *B*, original traces at the corresponding time points of (*A*). *C*, voltage-dependent hNav1.5 traces were recorded in a representative cell using the protocol in the inset, with control, 3 μM exenatide, and after washout. *D*, *I-V* relationships of hNav1.5 current in the absence or presence of 3 μM exenatide (n = 7; *p* < 0.05 or *p* < 0.01 *versus* control, paired Student’s *t* test). *E*, concentration–response curve of exenatide in inhibiting hNav1.5 current at −35 mV (n = 3–7). Symbols are the mean values of inhibitory effect in cells exposed to different concentrations of exenatide. Data points were fitted to the Hill equation.

The effects of exenatide on hNav1.5 current were determined in HEK 293 cells expressing human SCN5A. Figure 3*A* displays the time course of *I*_Nav1.5_ in a representative experiment with a 100-ms voltage step to −35 mV from −120 mV with control, 3 μM exenatide, and after washout. Exenatide gradually reduced *I*_Nav1.5_, which reached a steady state after approximately 7 min. The inhibition was partially reversed by washout. Figure 3*B* shows the original traces of the hNav1.5 currents at the corresponding time point of Figure 3*A*. Voltage-dependent hNav1.5 traces were recorded in a representative cell with control (approximately 5 min for initial phase), 3 μM exenatide (around 7 min for stable effect), and after washout (approximately 3 min) using the voltage protocol shown in the inset (Fig. 3*C*). Figure 3*D* illustrates the *I-V* relationships of hNav1.5 current density in the absence or presence of 3 μM exenatide, in which hNav1.5 current density was significantly inhibited by 3 μM exenatide at −50 to +50 mV (n = 7; *p* < 0.05 or *p* < 0.01 *versus* control). The concentration–response curve of exenatide for inhibiting hNav1.5 current was fitted by a Hill equation with IC_50_ of 3.30 μM and Hill coefficient of 1.9 (Fig. 3*E*).

In addition, the inhibitory effect of exenatide on late *I*_Nav1.5_ was measured by analyzing the current at 100 ms after the onset of the depolarizing step (24). Results showed that 3 μM exenatide also inhibited late *I*_Nav1.5_ (Fig. S3*A*); the late *I*_Nav1.5_ density was slightly reduced from −4.33 ± 1.88 pA/pF to −3.40 ± 0.94 pA/pF (Fig. S3 B; n = 5; *p* < 0.05).

### Blocking properties of hNav1.5 channel by exenatide

**Figure 4:**
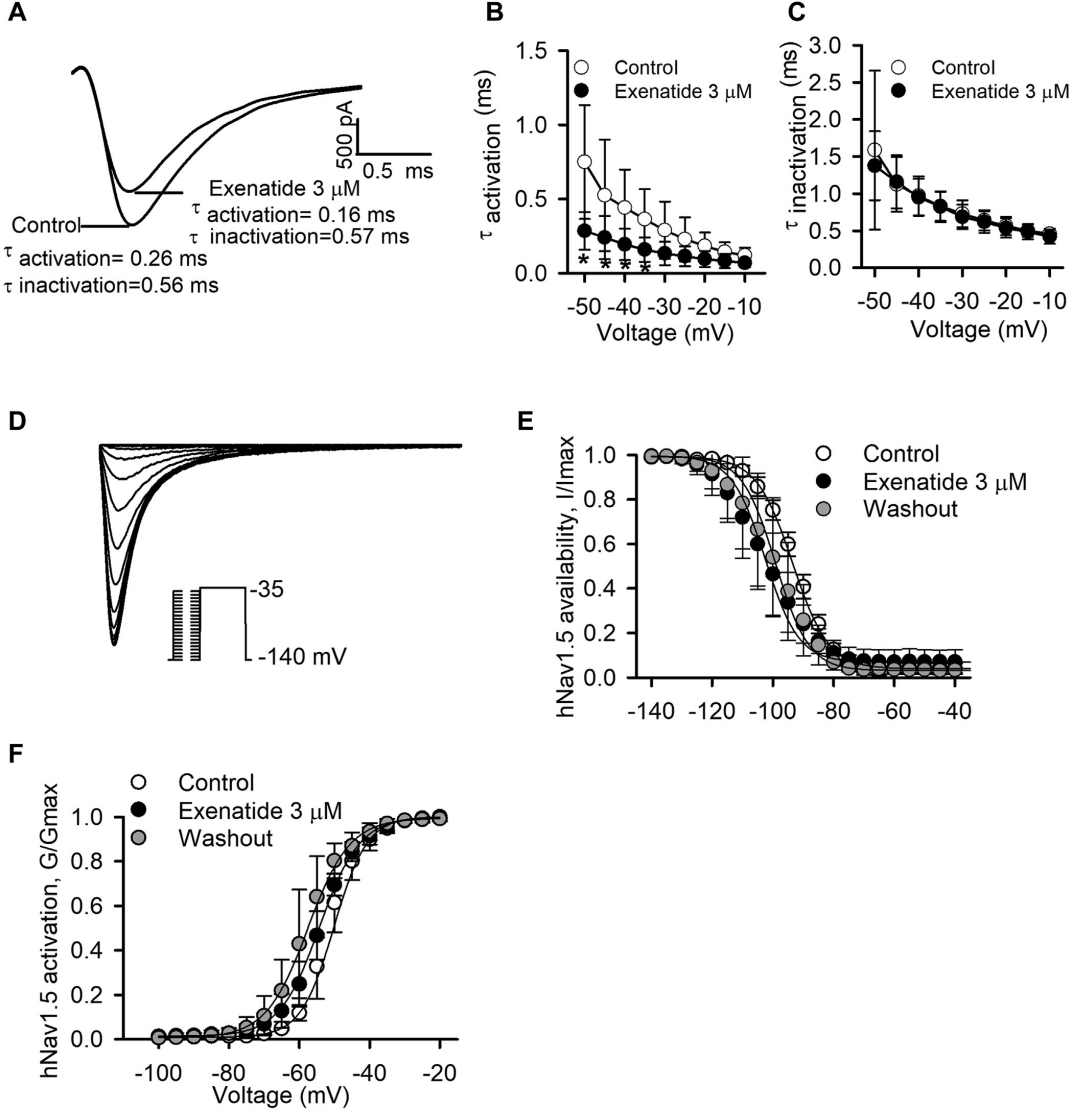
**Blocking properties of exenatide on hNav1.5 channel.***A*, *I*_Nav1.5_ recorded from −120 mV to −35 mV in control and after 3 μM exenatide treatment. *B*, mean values of activation time constant (τ _activation_) under control and after 3 μM exenatide treatment (n = 6; *p* < 0.05 *versus* control, non-paired Student’s *t* test). *C*, inactivation time constant (τ _inactivation_) under control and after 3 μM exenatide treatment (n = 6; *p* > 0.05 *versus* control, non-paired Student’s *t* test). *D*, voltage protocol and superimposed current for determining the availability (*I*/*I*_max_) of *I*_Nav1.5_. *E*, mean values of hNav1.5 availability in the absence or presence of 3 μM exenatide fitted to the Boltzmann equation (n = 6). *F*, mean values of hNav1.5 activation in the absence or presence of 3 μM exenatide fitted to the Boltzmann equation (n = 5).

Time-dependent kinetics of *I*_Nav1.5_ was assessed by analyzing the activation and inactivation processes with voltage step from −120 to −35 mV fitted to a monoexponential equation before and after 3 μM exenatide treatment (Fig. 4*A*). The activation time constant (Fig. 4*B*; n = 6, *p* < 0.05 at −50 to −35 mV), but not the inactivation time constant (Fig. 4*C*; n = 6), was reduced by exenatide. Therefore, exenatide accelerated time-dependent hNav1.5 channel activation, suggesting an open channel blocking property.

Voltage-dependent availability (I/Imax) of *I*_Nav1.5_ was determined as illustrated in Figure 4*D*, and the normalized availability curve was fitted by the Boltzmann equation (Fig. 4*E*) in the absence or presence of 3 μM exenatide. The *V*_1/2_ of *I*_Nav1.5_ availability was negatively shifted by 9.68 mV with 3 μM exenatide treatment (from −92.84 ± 0.75 mV for control to −102.52 ± 1.32 mV for exenatide; n = 6; *p* < 0.05), and recovered to −99.11 ± 1.23 mV after washout (n = 6), suggesting that the negative shift of *I*_Nav1.5_ availability was caused by the effect of exenatide. The slope factor value (κ) was not altered after exenatide treatment. G/Gmax of *I*_Nav1.5_ was determined from the *I-V* relationship for each cell and fitted to the Boltzmann equation (Fig. 4*F*). The *V*_1/2_ of *I*_Nav1.5_ activation was slightly shifted after 3 μM exenatide treatment (from −50.16 ± 0.35 mV for control to −54.25 ± 0.21 mV for exenatide; n = 5; *p* > 0.05), and further negatively shifted to −57.59 ± 0.26 mV after washout (n = 5). This result suggested that the negative shifts of *I*_Nav1.5_ activation were caused by the patch duration rather than the effect of exenatide.

To determine the potential effects of patch duration on hNav1.5 current, *I*_Nav1.5_ was recorded in cells treated with control after a stable current was reached (after 5 min of membrane rupture, initial phase) until an additional 15 min (Fig. S2*D*). The amplitude of peak *I*_Nav1.5_ was slightly decreased by 4.46 ± 1.01% (Fig. S2*E*; n = 3). These results indicated that the potential influence of patch duration on exenatide-induced reduction of peak *I*_Nav1.5_ amplitude is limited. It is demonstrated that the voltage dependence of *I*_Na_ activation and inactivation is affected by the patch time (25). Fig. S2, *F* and *G* illustrates the observation of the shifting rates of voltage-dependent kinetics in the same individual cell. The activation *V*_1/2_ and availability *V*_1/2_ of *I*_Nav1.5_ was shifted by −5.63 mV (Fig. S2*F*; n = 5; *p* > 0.05) and −4.02 mV (Fig. S2*G*; n = 5; *p* > 0.05), respectively. Fig. S4*A* shows the *V*_1/2_ availability of *I*_Nav1.5_ with exenatide was shifted to a more negative potential than those with the time control (n = 6; *p* < 0.05).

### Effects of exenatide on recovery from inactivation and use-dependence of hNav1.5 channel

**Figure 5:**
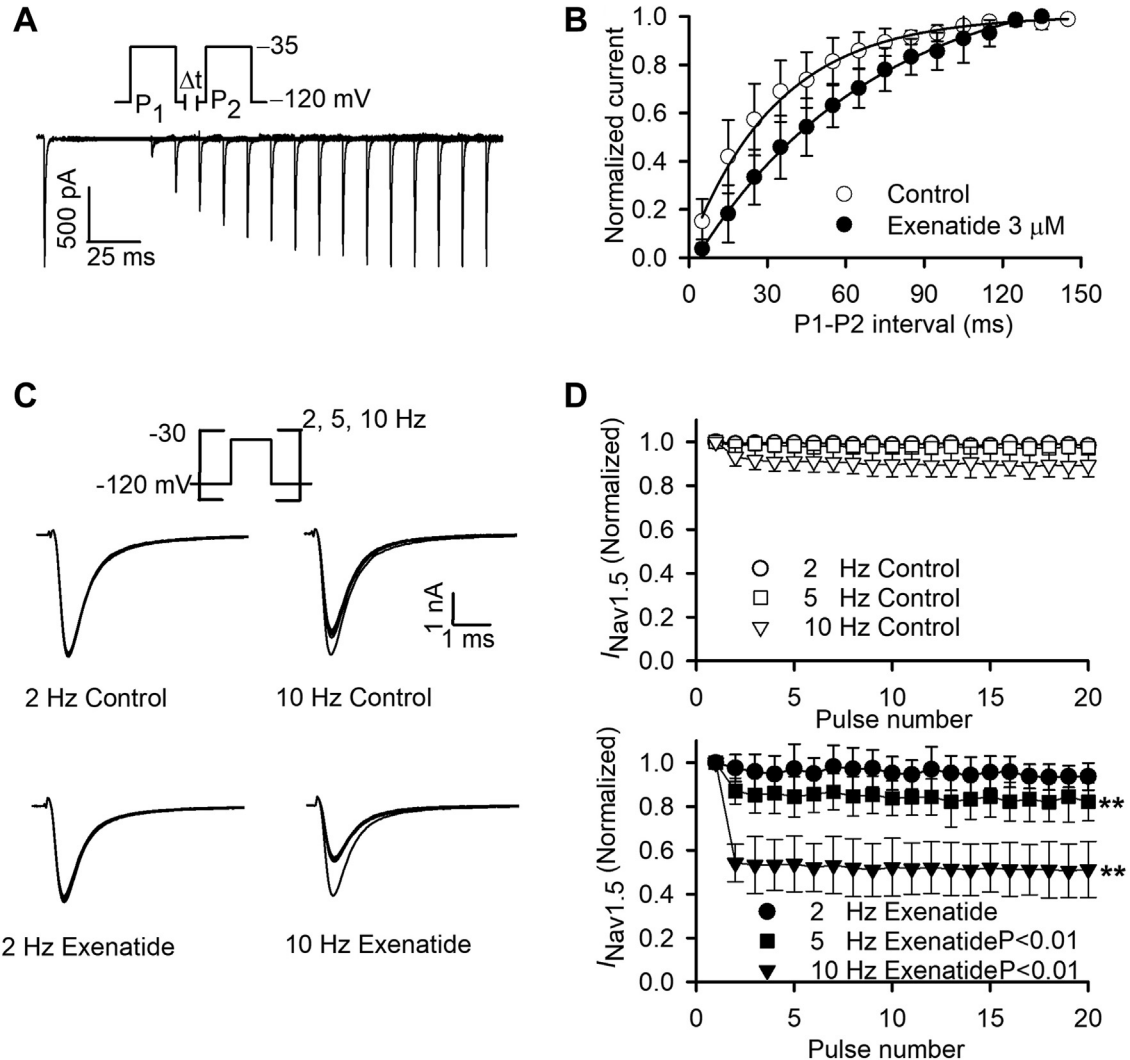
**Effects of exenatide on recovery from inactivation and use-dependence of hNav1.5 channel.***A*, protocol and typical current traces used to determine the recovery of *I*_Nav1.5_ from inactivation. *B*, the normalized current of *I*_Nav1.5_ plotted against the inter-pulse interval from inactivation with control and 3 μM exenatide. Recovery curve was fitted by the monoexponential function (n = 5). *C*, superimposed recordings obtained using 20 successive (30-ms) depolarizing pulses from −120 to −30 mV at 2 and 10 Hz before and after 3 μM exenatide treatment. *D*, normalized hNav1.5 current (normalized to first pulse) plotted against the number of pulses applied at 2, 5, and 10 Hz in the absence (*upper* panel) and presence (*lower* panel) of 3 μM exenatide (n = 5; *p* < 0.01, inhibition compared with control; 20th vs. First pulses, non-paired Student’s *t* test).

Superimposed current of recovery of *I*_Nav1.5_ from inactivation was determined with a paired-pulse protocol (Fig. 5*A*). *I*_Nav1.5_ recovery in the absence or presence of exenatide was complete and well-fitted by a monoexponential function (Fig. 5*B*). Fig. S2*H* shows the *I*_Nav1.5_ recovery under control with path time. The average time constants of *I*_Nav1.5_ recovery from inactivation increased by 33.85 ms, from 34.18 ± 1.45 ms for the initial phase to 68.03 ± 2.67 ms after 3 μM exenatide (Figs. 5*B* and S4*B*; n = 5; *p* < 0.01). However, the recovery time constant slightly increased by 8.13 ms with time control (Fig. S4*B*; n = 6; *p* > 0.05). The results indicated that exenatide slowed the recovery of *I*_Nav1.5_ from inactivation.

The slowed recovery of *I*_Nav1.5_ from inactivation by exenatide implied more inactivated state channels at a high frequency of stimulation. Therefore, the use-dependent inhibition of *I*_Nav1.5_ by exenatide was examined. The superimposed *I*_Nav1.5_ showed that the use-dependent inhibition increased after 3 μM exenatide treatment when pulsed at 10 Hz (Fig. 5*C*). The normalized *I*_Nav1.5_ at 2, 5, and 10 Hz was plotted against pulse number with control and 3 μM exenatide (Fig. 5*D*). The use-dependent inhibition of *I*_Nav1.5_ increased from 3 ± 2.26% for control to 17.88 ± 8.58% for exenatide at 5 Hz (n = 5; *p* < 0.01 *versus* control), and increased from 10.61 ± 5.42% for control to 48.73 ± 12.81% for exenatide at 10 Hz (n = 5; *p* < 0.01 *versus* control). These results indicated that exenatide is an open-channel blocker of hNav1.5 channel.

### Effects of exenatide on action potential and other cardiac ionic currents in adult rat atrial myocytes

**Figure 6:**
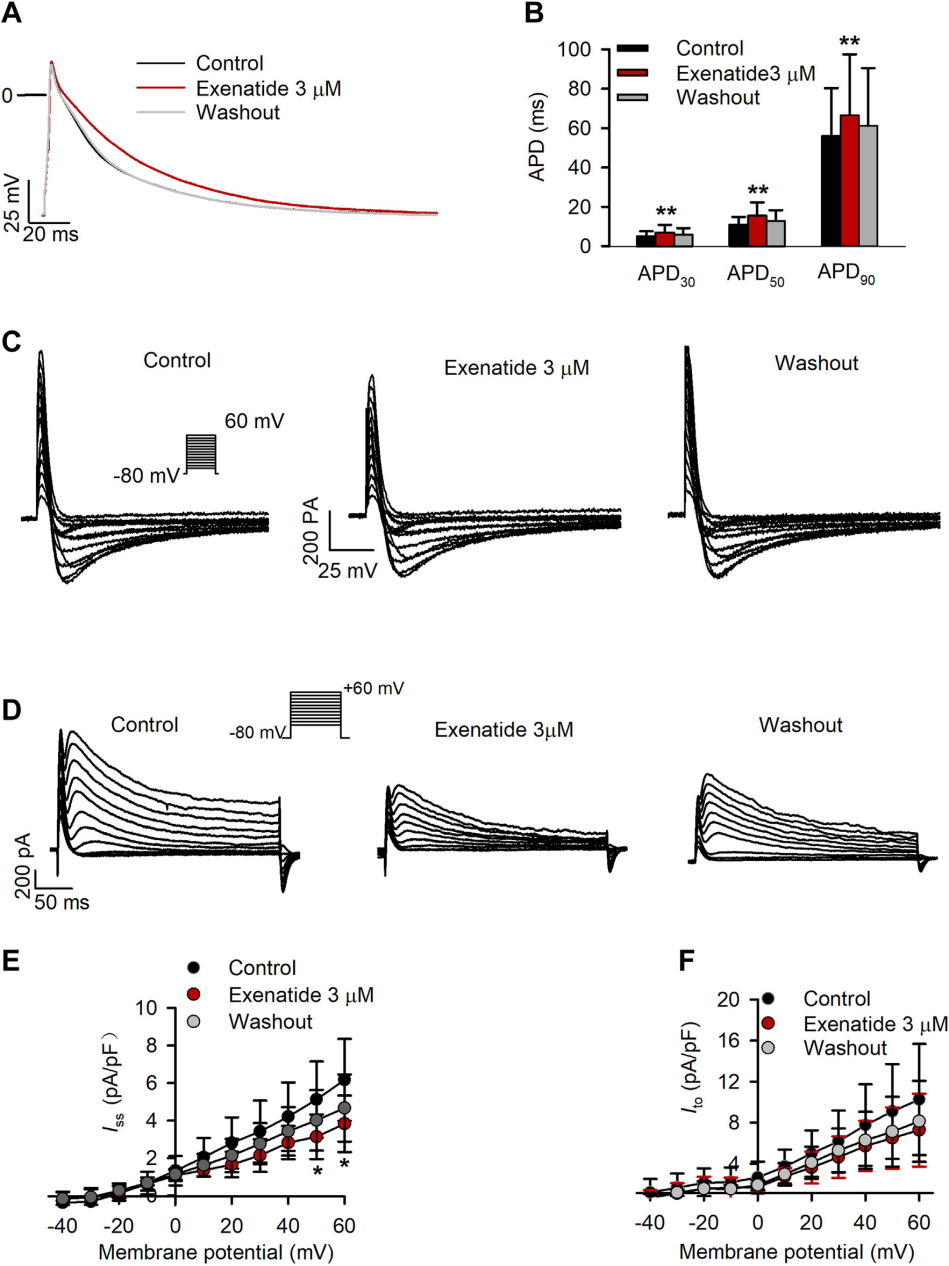
**Effects of exenatide on action potential and other cardiac ionic currents in adult rat atrial myocytes.***A*, action potentials recorded at 1 Hz in the absence or presence of 3 μM exenatide in a representative cell. *B*, exenatide 3 μM prolonged APD at 30%, 50%, and 90% repolarization (APD_30,_ APD_50,_ and APD_90_; n = 8; *p* < 0.01 *versus* control; paired Student’s *t* test). *C*, representative voltage-dependent *I*_Ca,L_ recorded with 300-ms voltage steps to between −60 and +60 mV from −80 mV in the absence or presence of 3 μM exenatide. *D*, representative voltage-dependent *I*_ss_ and *I*_to_ recorded with 300-ms voltage steps to between −40 and +60 mV from −80 mV in the absence or presence of 3 μM exenatide. *E*, *I*-*V* relationships of *I*_ss_ in the presence of control, 3 μM exenatide, and drug washout (n = 4; *p* < 0.05 *versus* control; paired Student’s *t* test). *F*, *I*-*V* relationships of *I*_to_ under control, in the absence of 3 μM exenatide and drug washout (n = 4; *p* > 0.05 *versus* control; paired Student’s *t* test).

The inhibition of *I*_Kv1.5_ and *I*_Nav1.5_ by exenatide suggested that it may prolong the APD in isolated adult rat atrial myocytes. Therefore, we recorded action potentials in a current clamp mode. Figure 6*A* illustrates action potentials recorded at 1 Hz in a representative adult rat atrial myocyte, in the absence or presence of 3 μM exenatide. The action potential duration at 30% (APD_30_), 50% (APD_50_), and 90% (APD_90_) repolarization was increased after exenatide treatment (Fig. 6*B*; n = 8; *p* < 0.05 *versus* control).

The potential effects of exenatide on other cardiac ionic currents, including L-type calcium current (*I*_Ca,L_), sustained K^+^ current (*I*_ss_), and transient outward K^+^ current (*I*_to_) were further determined in adult rat atrial myocytes, as previously described (26). Interestingly, *I*_Ca,L_ was not affected by 3 μM exenatide (Fig. 6*C*; n = 3); however, the voltage-dependent *I*_to_ and *I*_ss_ were decreased by 3 μM exenatide (Fig. 6*D*). Significant *I*_s__s_ density reduction was observed at test potentials of +50 to +60 mV (Fig. 6*E*; n = 4; *p* < 0.05 *versus* control), while exenatide-induced inhibition of *I*_to_ was not as significant as that of *I*_s__s_ (Fig. 6*F*; n = 4; *p* > 0.05 *versus* control). In addition, exenatide at 3 μM had no inhibitory effect on inward rectifier K^+^ current (*I*_K1_) in isolated adult rat ventricular myocytes (data not shown).

### Exenatide reduces AF susceptibility in isolated rat hearts

**Figure 7:**
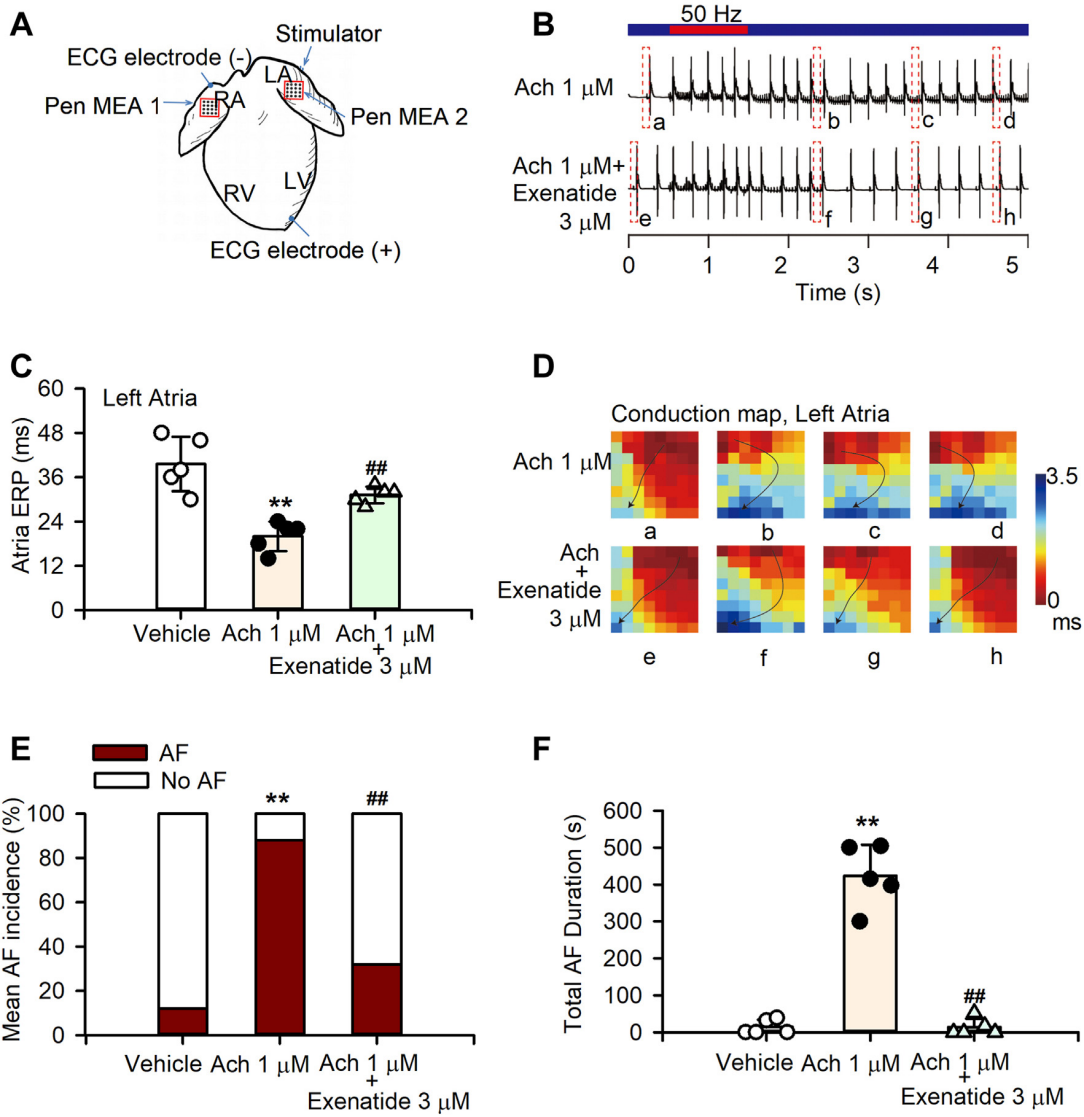
**Effects of exenatide on AF susceptibility in isolated rat hearts.***A*, schematic illustration of the locations of electrical mapping and ECG electrodes in the model heart. *B*, representative ECG recordings (5-s duration) from an isolated rat heart perfused with acetylcholine or co-perfused with exenatide, before and after burst pacing at 50 Hz for 1 s. *C*, mean values of left atrial ERP in isolated rat hearts (n = 5 hearts/group; ^∗∗^*p* < 0.01 *versus* vehicle, ^##^*p* < 0.01 *versus* 1 μM acetylcholine; ANOVA, Tukey's post hoc test). *D*, conduction maps of *left* atria with acetylcholine or combined with 3 μM exenatide co-perfusion at the corresponding time points of (*B*). Arrows show the direction of spread excitement and indicate the pacing site. *E*, mean AF incidence in isolated rat hearts (n = 5 hearts, five repeats per heart; ^∗∗^*p* < 0.01 *versus* vehicle, ^##^*p* < 0.01 *versus* 1 μM acetylcholine; Fisher's exact test, Bonferroni correction). *F*, total AF duration in isolated rat hearts (n = 5 hearts, five repeats per heart; ∗∗*p* < 0.01 *versus* vehicle, ^##^*p* < 0.01 *versus* 1 μM acetylcholine; ANOVA, Tukey's post hoc test). Ach stands for acetylcholine in this figure.

Figure 7*A* illustrates the locations of mapping and ECG electrodes in the model heart. The susceptibility to AF was markedly increased and AF episodes of long duration were observed in acetylcholine-treated rat hearts; however, co-treatment with 3 μM exenatide had a notable effect in preventing AF maintenance and resulted in a shorter duration of AF episodes (Fig. 7*B*). Left atria ERP decreased from 39.6 ± 7.4 to 20 ± 4 ms in acetylcholine-treated hearts (n = 5, *p* < 0.01 *versus* vehicle), which was rescued to 31.2 ± 2.28 ms by exenatide co-treatment (Fig. 7*C*, n = 5, *p* < 0.01 *versus* acetylcholine alone). Figure 7*D* shows the representative conduction map in the left atria of the rat heart at the corresponding time points of Figure 7*B*. Pacing triggered atrial ectopy and re-entry in acetylcholine-treated rat hearts; however, co-perfusion with 3 μM exenatide markedly prevented the disorganized atrial conduction (Fig. 7*D*). The mean AF incidences per heart increased from 12 ± 17.89% to 88 ± 10.95% under acetylcholine treatment (n = 5, *p* < 0.01 *versus* vehicle), whereas they reduced to 32 ± 46.04% after co-perfusion with 3 μM exenatide (n = 5, *p* < 0.05 *versus* acetylcholine alone) (Fig. 7*E*). The total AF duration per heart decreased from 423.26 ± 84.27 s under acetylcholine treatment to 14.0 ± 22.79 s after 3 μM exenatide co-perfusion (Fig. 7*F*, n = 5, *p* < 0.01 *versus* acetylcholine alone). These results indicated that 3 μM exenatide decreases AF susceptibility in isolated rat hearts.

### Exenatide decreases AF susceptibility in rats

**Figure 8:**
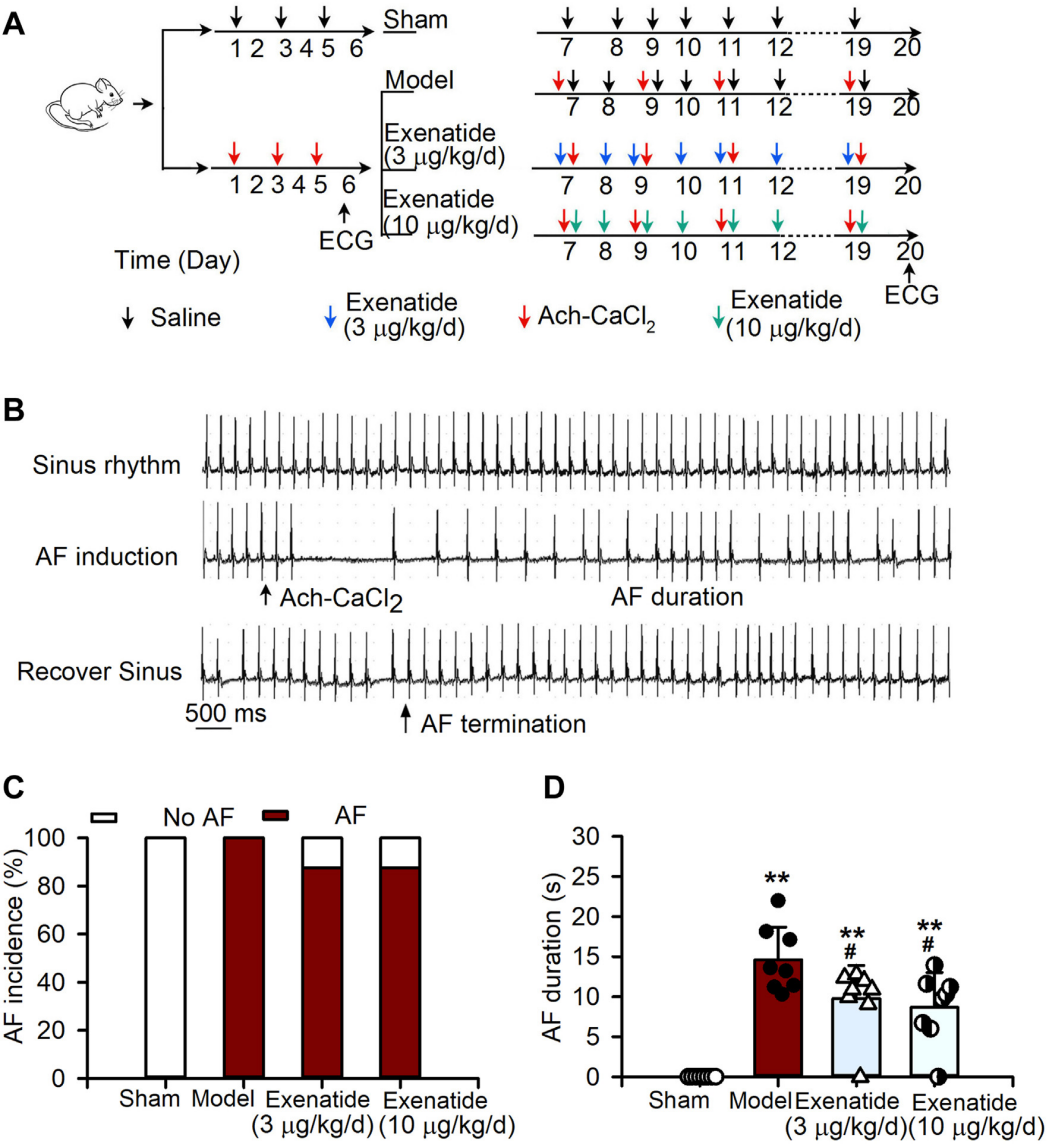
**Effects of exenatide on AF susceptibility in rats.***A*, experimental design and grouping of rats. *B*, representative ECG recordings of normal sinus rhythm, induction and maintenance of AF, and recovery to normal sinus rhythm in a rat. *C*, percentage of AF incidences in rats. *D*, mean AF duration in rats (n = 8 rats per group; ∗∗*p* < 0.01 *versus* Sham, ^#^*p*< 0.05 *versus* Model; Kruskal–Wallis test, Mann–Whitney U test, FDR correction). Ach stands for acetylcholine in this figure.

The schematic design of a rat AF susceptibility model for evaluating the exenatide effect is depicted in Figure 8*A*. Figure 8*B* shows the representative ECG recordings during the sinus rhythm, after the injection of acetylcholine-CaCl_2_ to induce AF, as well as recovery to the sinus rhythm in a rat. AF was never induced in the sham group (0/8 rats); but was 100% induced in the model group (8/8 rats), and its incidence was reduced to 87.5% (seven-eighths rats) in the 3 or 10 μg/kg/day exenatide groups (Fig. 8*C*). The average AF duration decreased from 14.61 ± 4.07 s in model rats to 9.76 ± 4.13 s or 8.67± 4.35 s in the 3 or 10 μg/kg/day exenatide groups, respectively (Fig. 8*D*, n = 8, *p* < 0.05 *versus* model). These results indicated that exenatide decreases AF susceptibility in rats.

## Discussion

The incidence of AF globally is steadily rising, and the condition is associated with an increased risk of mortality and morbidity. However, the efficacy of current therapies is suboptimal (27). Studies have shown that exenatide has the potential to preserve cardiac function and reduce infarct size (20, 21). Additionally, exenatide can protect endothelial dysfunction during ischemia-reperfusion by opening the ATP-sensitive potassium (K_ATP_) channels (28). However, the present study provided the novel pharmacological effect that exenatide inhibits hKv1.5 and hNav1.5 currents and reduces AF susceptibility in isolated rat hearts and in rats. Nonetheless, the inhibitory effect of exenatide on hKv1.5 and hNav1.5 channels was not significantly affected in the presence of a GLP-1 receptor antagonist. This finding implied that the inhibitory effect of exenatide on these cardiac channels may be independent of GLP-1 receptor-mediated cellular responses, as depicted in Fig. S5.

State-dependent inhibition is classified into open and/or closed states. We offered several lines of evidence suggesting that exenatide inhibits the hKv1.5 channel current in a closed-state-dependent manner. In general, open-state blockers inhibit the steady-state amplitude more than the peak amplitude (29); however, exenatide suppressed both peak and steady-state currents of hKv1.5 to a similar degree. Moreover, when compared to the mean values for the initial tonic blocking, only a limited component of the open channel blocking effect of exenatide on *I*_Kv1.5_ was observed. Furthermore, the deceleration of the activation time course caused by exenatide was consistent with the behavior of closed channel blockers (30). In addition, use- and frequency-dependent inhibition of hKv1.5 was not observed with 3 μM exenatide, which constitutes strong evidence of closed-channel inhibition (31).

With regard to sodium channels, voltage-dependent blockade in the open state is the more important drug-channel interaction for antiarrhythmic drugs (32). It was found that exenatide potently and reversibly blocked hNav1.5 currents in a voltage- and concentration-dependent manner. Moreover, consistent with the effects of various antiarrhythmic compounds (33, 34), exenatide inhibited *I*_Nav1.5_ in a use-dependent manner when pulsed at 5 and 10 Hz. The use-dependence of exenatide may arise from the need for hNav1.5 channel to open for the drug to reach its high-affinity receptor within the open pore and also implies that exenatide has potential applications as an anti-fibrillation agent (35). Additionally, exenatide was found to shift the voltage dependence of steady-state inactivation toward a more hyperpolarizing direction and slowed the recovery of hNav1.5 channel from inactivation. These results supported the notion that exenatide functions as an inhibitor of the open hNav1.5 channel by blocking both its open and inactivated states, similar to other sodium channel modulators, such as hesperetin (34).

Although sole *I*_Kur_ inhibitors have shown promise in cellular experiments and animal models by prolonging atrial APD and atrial ERP without affecting ventricular repolarization, their anti-AF efficacy in clinical studies is limited. However, there might be some potential for anti-AF activity by blocking multiple ion channels (12). For instance, vernakalant, registered by the European authorities for conversion of recent AF, inhibits *I*_Kur_ in a frequency-dependent manner and suppresses the upstroke velocity of the action potential, which is relevant to the inhibition of *I*_Nav1.5_ (36, 37).

Here, exenatide was identified to function as an inhibitor of the open hNav1.5 channel and an inhibitor of the atrial-specific hKv1.5 channel in the closed state. In addition, exenatide did not inhibit hKv1.5 and hNav1.5 currents through GLP-1 receptor-dependent signaling. Moreover, exenatide exhibited no effect on *I*_Ca,L_ in rat atrial myocytes and *I*_K1_ in rat ventricular myocytes (data not shown). Furthermore, exenatide was revealed to prevent disorganized conduction in isolated rat hearts and prolong APD in rat atrial myocytes. Finally, exenatide reduced the frequency and duration of AF in isolated rat hearts and in rats.

In summary, the present study demonstrated that exenatide inhibits hKv1.5 channels and hNav1.5 channels independent of GLP-1 receptors *in vitro*, and reduces AF susceptibility in isolated rat hearts and rats. Exenatide preferentially inhibits the hKv1.5 channel by binding to its closed state. However, it functions as an inhibitor of the open hNav1.5 channel by blocking both its open and inactivated states. The combined effects of exenatide on *I*_Kv1.5_ and *I*_Nav1.5_ likely contribute to its ability to reduce AF susceptibility in isolated rat hearts and rats.

The major limitation of the present study was that the affinity of exenatide for hKv1.5 and hNav1.5 channels was much lower than that reported for GLP-1 receptors. In clinical use, exenatide has been demonstrated to maintain plasma concentrations ranging from 50 to100 PM (38), while in our *in vitro* experiments, exenatide in the low micromolar range inhibited hKv1.5 and hNav1.5 currents, which is much higher than the physiological lever. Therefore, whether it is feasible to increase exenatide *in vivo* in patients with AF to high enough concentrations to inhibit hKv1.5 and hNav1.5 channels without inducing side effects is unclear. This will be confirmed in future studies.

## Experimental procedures

### Chemicals and reagents

Acetylcholine, CaCl_2_, N-methyl-D-glucamine, and bovine serum albumin were purchased from Sigma-Aldrich. Exenatide (Byetta) was purchased from Baxter Pharmaceutical Solutions LLC. Type II collagenase was purchased from Worthington Biochemical Corp. Deionized water used in all experiments was purified with the Milli-Q academic water purification system. Other chemical reagents were of analytical grade and purchased from local chemical suppliers.

### Cell culture

HEK 293 cells stably expressing human cardiac Nav1.5 (*hSCNA5*) and human Kv1.5 (*hKCNA5*) were kindly provided by Prof Guirong Li (Nanjing Amazigh Pharma Limited). Cells were cultured in Dulbecco’s modified Eagle’s medium containing 600 μg/ml geneticin (G418, Sigma-Aldrich) and 10% fetal bovine serum (Sigma-Aldrich) at 37 °C in an incubator with 5% CO_2_ and passaged every 2 days.

### Animal use and care

Considering the higher prevalence and incidence of AF in men than in women (27), Sprague–Dawley (SD) rats (**♂**, 220 ± 10 g) were used in this study. All animal experiments in this study were approved by the Ethics Committee of Jiangsu Province Hospital on Integration of Chinese and Western Medicine (AEWC-20210716-159).

### Single cardiomyocyte preparation

Cardiomyocytes were enzymatically dissociated as previously described (39). Rats were anesthetized with 3% pentobarbital sodium (50 mg/kg) injected intraperitoneally. The heart was quickly excised and mounted on a Langendorff apparatus. The heart was retrogradely perfused for 5-min at 37 °C with oxygenated Tyrode solution, followed by a nominally Ca^2+^–free Tyrode solution for 5 to 10 min, and a 20 to 30 min perfusion with nominally Ca^2+^ free solution containing 0.5 mg/ml type II collagenase and 1 mg/ml bovine serum albumin. Subsequently, atrial tissue was removed from the softened heart and gently pipetted. Cells were suspended in a high K^+^ solution containing (in mM) 10 KCl, 120 K-glutamate, 10 KH_2_PO_4_, 1.8 MgSO_4_, 10 taurine, 10 HEPES, 0.5 EGTA, 20 glucose, and 10 mannitol, with pH adjusted to 7.3 using KOH. Isolated myocytes were kept at room temperature (22 ± 1 °C) in the medium for at least 1 h before the experiment. A small aliquot of the solution containing the isolated cells was placed in a perfusion chamber mounted on an inverted microscope (IX-73; Olympus, Tokyo, Japan) and superfused with Tyrode solution after cells attached to the chamber bottom (2 ml/min). Only quiescent rod-shaped cells showing clear cross-striations were used.

### Solution

For hKv1.5 current recording, a pipette solution containing (in mM) 10 NaCl, 120 KCl, 1 EGTA, 10 HEPES, and 1.1 MgCl_2_ (pH adjusted to 7.2 with KOH) was used. The pipette had a tip resistance of 3 to 4 MΩ when filled with the pipette solution. The bath solution contained (in mM) 140 NaCl, 4.7 KCl, 1.1 MgCl_2_, 2 CaCl_2_, 10 HEPES, and 10 glucose (pH adjusted to 7.4 with NaOH). For hNav1.5 current recording, a pipette solution containing (in mM) 10 NaCl, 1 EGTA, 10 HEPES, and 140 CsF (pH adjusted to 7.3 with CsOH) was used. The resistance of the pipettes ranged from 2 to 4 MΩ when filled with the pipette solution. The bath solution contained (in mM) 70 NaCl, 70 CsCl, 3 KCl, 1 MgCl_2_, 1 CaCl_2_, 10 HEPES, and 10 glucose (pH adjusted to 7.3 with NaOH). The Tyrode solution contained (in mM) 136 NaCl, 5.4 KCl, 1.0 Mg Cl_2_, 2.0 CaCl_2_, 0.33 NaH_2_PO4, 10.0 glucose, and 10 HEPES, with pH adjusted to 7.3 with NaOH. For atrial tissue wash, Ca^2+^ was omitted. For recording the action potential, myocytes were perfused with Tyrode solution (current-clamp mode). The pipette solution contained (in mM) 10 NaCl, 120 KCl, 2 MgCl_2_, 5 Na_2_-ATP, 10 EGTA, 10 HEPES, and 0.1 GTP (pH adjusted to 7.2 with KOH). For *I*_Ca,L_, myocytes were perfused with the Na^+^-free N-methyl-D-glucamine (NMDG) solution, containing (in mM) 136 NMDG, 5.4 CsCl, 1.0 MgCl_2_, 2.0 CaCl_2_, 2.0 NaH_2_PO_4_, 10 glucose, and 10 Hepes (pH adjusted to 7.3 with NMDG). The pipette solution contained (in mM) 20 CsCl, 110 cesium aspartate, 1.0 MgCl_2_, 10 HEPES, 10 EGTA, 0.1 GTP, and 5 Mg_2_ATP (pH adjusted to 7.2 with CsOH). For *I*_to_ and *I*_ss_, myocytes were perfused with a Na^+^-free (NMDG replacement) Tyrode solution, with BaCl_2_ (500 μM) and CdCl_2_ (200 μM) to block *I*_K1_ and *I*_Ca_. The pipette solution contained (in mM) 20 KCl, 110 K-aspartate, 1.0 MgCl_2_, 10 HEPES, 5 EGTA, 0.1 GTP, 5 Na_2_-phosphocreatine, and 5 Mg_2_ATP (pH adjusted to 7.2 with KOH) (26, 40, 41).

### Whole-cell patch recording

Cells were seeded in a perfusion chamber mounted on an inverted microscope (IX-73; Olympus) and superfused with bath solution after they attached to the chamber bottom (2 ml/min). Glass pipettes were pulled using a Brown–Flaming puller (P-97; Sutter Instrument, Novato, CA, USA), and tips were heat-polished. Recordings were performed using an Axopatch 200 B amplifier, a Digidata 1200 interface, and pClamp 10.5 software (Molecular Devices, Sunnyvale, CA, USA). The collected data were filtered at 5 Hz and sampled at 10 kHz. All patch-clamp recording experiments were conducted at room temperature (22 ± 1 °C).

### Electrical mapping of rat hearts

Rats were anesthetized with 3% pentobarbital sodium (50 mg/kg) injected intraperitoneally. The heart was quickly isolated and placed in Krebs–Henseleit (KH) solution containing (in mM) 119 NaCl, 4 KCl, 1.8 CaCl_2_, 1 MgCl_2_, 1.2 NaH_2_PO_4_, 25 NaHCO_3_, and 10 glucose at 4 °C to washout residual blood and to remove residual lung tissues. The aorta was cannulated and connected to a Langendorff perfusion system filled with KH solution at 37 °C and gassed with 5% CO_2_ and 95% O_2_. Epicardial activating electrical mapping was recorded simultaneously using two multi-electrode arrays with 64 electrodes on the surface of the left and right atria. ECG electrodes were placed on the right atria and apex of the heart. A stimulator was placed on the left atrium to assess AF inducibility and duration. The stimulation order was from the left atrium to the right atrium. AF was initiated by perfusing 1 μM acetylcholine for 3 min and stimulated by burst pacing at 50 Hz for 1 s. Subsequently, 3 μM exenatide was added to the perfusion solution for 30 min, followed by pacing at 50 Hz for 1 s. The pacing was performed five times with 30-s intervals. AF was defined as rapid and irregular atrial response longer than 1 s. AF inducibility was defined as the percentage of burst pacing leading to AF episodes. Atrial ERP was determined using an extra stimulus protocol (S1-S2; 10 S1 stimuli at 6 Hz followed by a premature S2 stimulus ranging from 80 to 10 ms). The dose of exenatide used in this study was close to the IC_50_ values of exenatide on *I*_Kv1.5_ and *I*_Nav1.5_ and ensured a stable anti-AF effect in isolated rat hearts. Data were recorded using a multichannel system (EMS64-USB-1003; MappingLab Ltd) and analyzed using the EmapScope 5.0 software (MappingLab Ltd).

### Animal experiments

The effect of exenatide on AF susceptibility in rats was studied by injecting acetylcholine and CaCl_2_ (42, 43). Forty rats were randomly assigned to two groups: rats in group 1 (n = 10) received an intravenous (i.v.) injection of saline into the tail vein every 2 days, three times. Rats in group 2 (n = 30) received an i.v. injection of 1 ml/kg acetylcholine-CaCl_2_ (5 mg CaCl_2_ and 33 μg acetylcholine/ml) every 2 days, three times. Before administration, rats were anesthetized using 2.5% isoflurane with an oxygen flow of 4 L/min. On day 7, saline was administered to group 1 rats (i.v.), whereas the acetylcholine-CaCl_2_ solution was administered to group 2 rats (i.v.) to select drug-sensitive rats using lead II electrocardiogram (ECG) monitoring with a BL-420 biology function laboratory system (TME Technology Co).

Subsequently, group 1 rats with normal ECG were selected as the sham group (Sham, n = 8), and 24 rats in group 2 exhibiting drug sensitivity were selected and randomly assigned to the following groups: model group, wherein rats received i.v. administration of 1 ml/kg acetylcholine-CaCl_2_ every 2 days, seven times (Model, n = 8); exenatide low-dose group, wherein rats received a daily intraperitoneal (i.p.) injection of 3 μg/kg/d exenatide and i.v. administration of acetylcholine-CaCl_2_ every 2 days, seven times (Exenatide 3 μg/kg/d, n = 8); and exenatide high-dose group, wherein rats received a daily i.p. injection of 10 μg/kg/d exenatide and i.v. administration of acetylcholine-CaCl_2_ every 2 days, seven times (Exenatide 10 μg/kg/d, n = 8). Exenatide was dissolved in saline. The dose of exenatide used in this study was based on the results of published studies that demonstrated potent cardioprotection in rats (21, 44). The sham and model group rats were administered an i.p. injection of an equal volume of saline. On day 20, lead II ECG was monitored during the period before drug administration and after f-wave appearance. At study completion, rats were euthanized via cervical dislocation after CO_2_-induced unconsciousness.

### Data acquisition and analysis

Nonlinear curve fitting was performed using OriginPro 8.5 and SigmaPlot 12.5. Data were expressed as the mean ± SD and analyzed with GraphPad Prism 8.0. The comparison between two groups was assessed by Student’s *t* test for non-paired replicates or paired Student’s *t* test. When comparing three or more parametric data sets, AVOVA with Tukey's post hoc test was used. If the normal distribution assumption was not met, the Kruskal–Wallis test and Mann–Whitney U test with FDR correction were utilized. Fisher's exact test was used for the comparison of Boolean variables with Bonferroni correction. Results with *p* < 0.05 were considered statistically significant.

## Data availability

The data supporting the findings of this study are available within the article. The data not shown can be obtained from the corresponding author on reasonable request.

## Supporting information

This article contains supporting information.

## Conflicts of interests

The authors declare that they have no conflicts of interest with the contents of this article.
