# Supplementary material for: Exenatide reduces atrial fibrillation susceptibility by inhibiting hKv1.5 and hNav1.5 channels
Source: J Biol Chem. 2024 Apr 16;300(5):107294. doi: 10.1016/j.jbc.2024.107294 (PMC11109313; doi:10.1016/j.jbc.2024.107294)
Supplement: Supporting Figures S1–S5 [file mmc1.docx]

**Supporting Information**

**I. Supplementary Methods**

GLP-1R antagonist (GLP-1Ra, Cat# T15387) was purchased from TargetMol (Shanghai, China), and prepared as 10 mM stock solutions in dimethyl sulfoxide. The stock was divided into aliquots and stored at -20 °C.

**II. Supplemental figures**

**
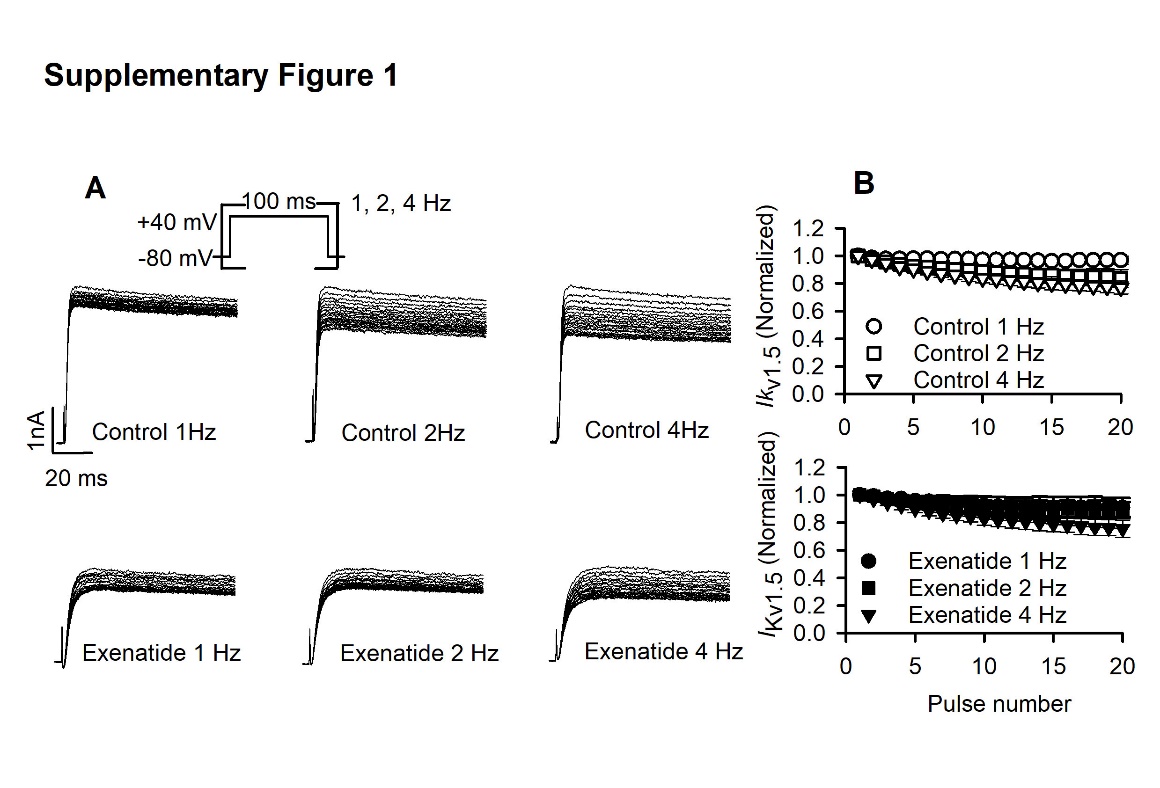
**

**Figure S1. Use (frequency) independence inhibition of hKv1.5 current by exenatide.** A. Superimposed recordings obtained using 20 successive (100-ms) depolarizing pulses from -80 to +40 mV at 1, 2, and 4 Hz before and after 3 μM exenatide treatment. B. Normalized hKv1.5 current (normalized to 1^st^ pulse) plotted against the number of pulses applied at 1, 2, and 4 Hz in the absence (upper panel) and presence (lower panel) of 3 μM exenatide (n=4; P > 0.05, inhibition compared with control; 20^th^ vs. 1^st^ pulses, non-paired Student’s *t*-test).

**
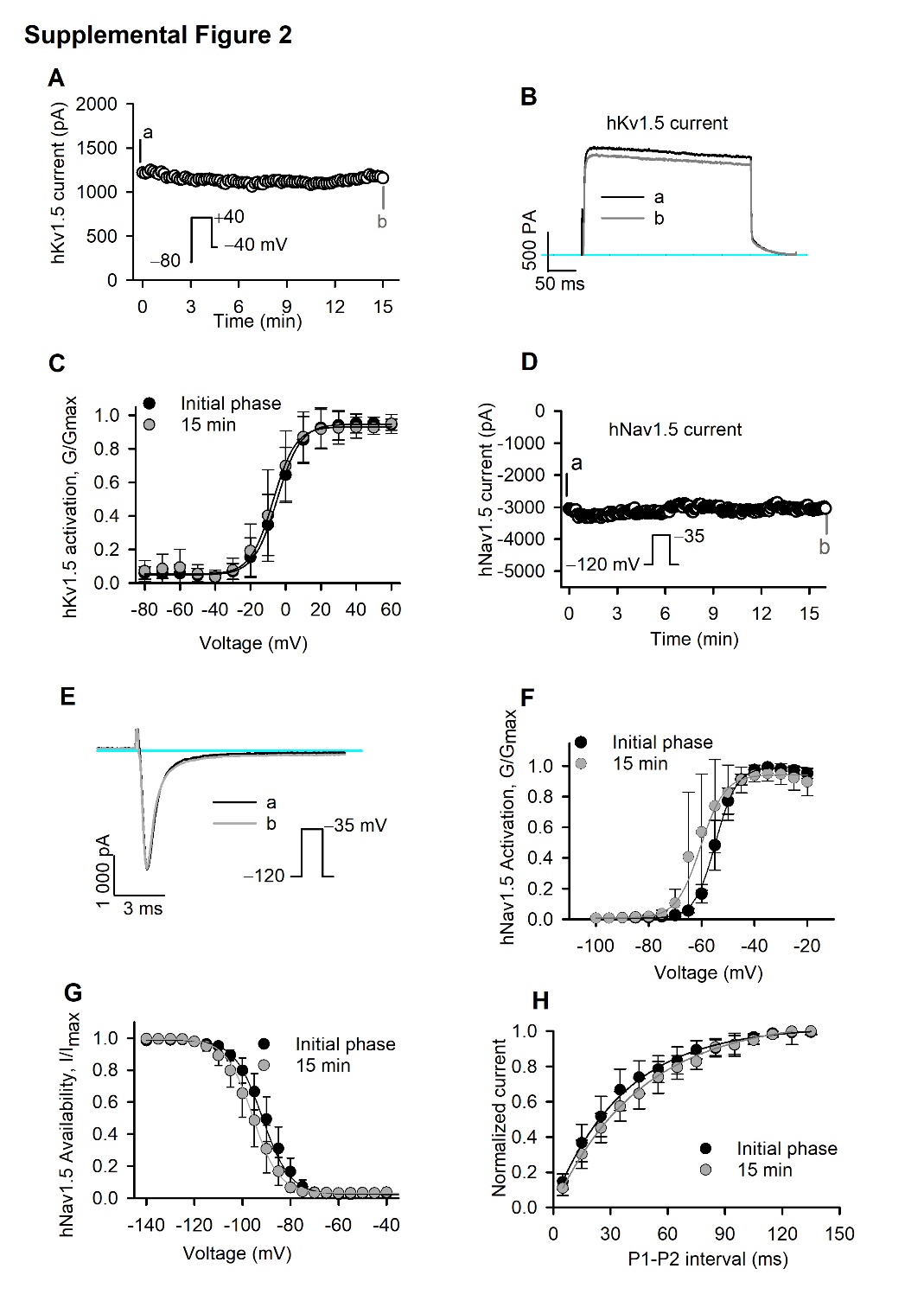
**

**Figure S2. Vehicle control experiments performed on hKv1.5 and hNav1.5 currents.** A. Time course of hKv1.5 current. B. Original hKv1.5 current traces at the corresponding time points of (A). C. Normalized hKv1.5 tail (G/Gmax) variables with the initial phase and at an additional 15 min fitted to the Boltzmann equation (n=6). D. Time course of hNav1.5 current. E. Original hNav1.5 current traces at the corresponding time points of (D). F. Mean values of hNav1.5 activation at the initial phase and at an additional 15 min fitted to the Boltzmann equation (n=5).G. Mean values of hNav1.5 availability at the initial phase and at an additional 15 min fitted to the Boltzmann equation (n=5). H. The normalized current of *I*_Nav1.5_ plotted against the inter-pulse interval from inactivation with the initial phase and at an additional 15 min. Recovery curves were fitted to a monoexponential function (n=6).


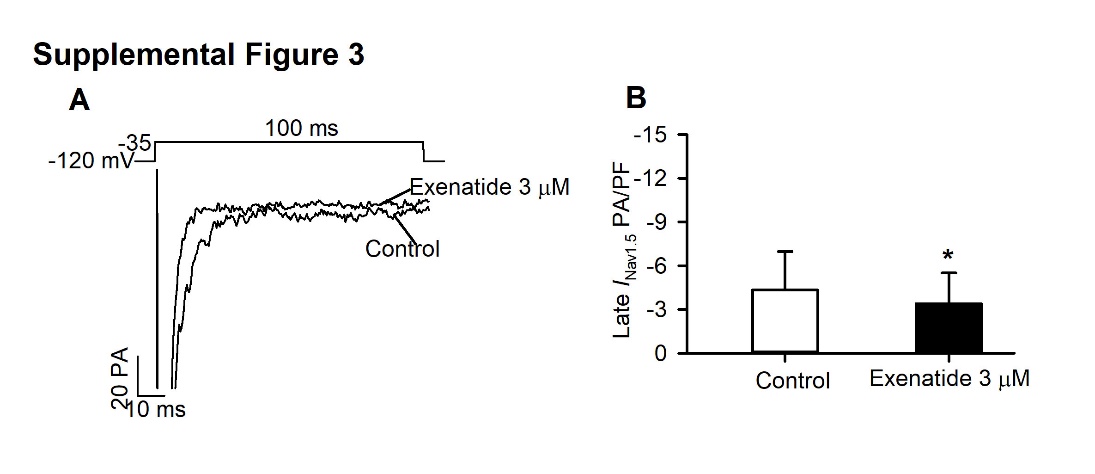


**Figure S3. Effects of exenatide on late *I*_Nav1.5_.** A. Representative currents of late *I*_Nav1.5_ under control and in the presence of 3 μM exenatide with 100-ms voltage depolarization steps from -120 to -35 mV. B. Mean values of late *I*_Nav1.5_ density at -35 mV (n=5; P < 0.05, paired Student’s *t*-test).

**
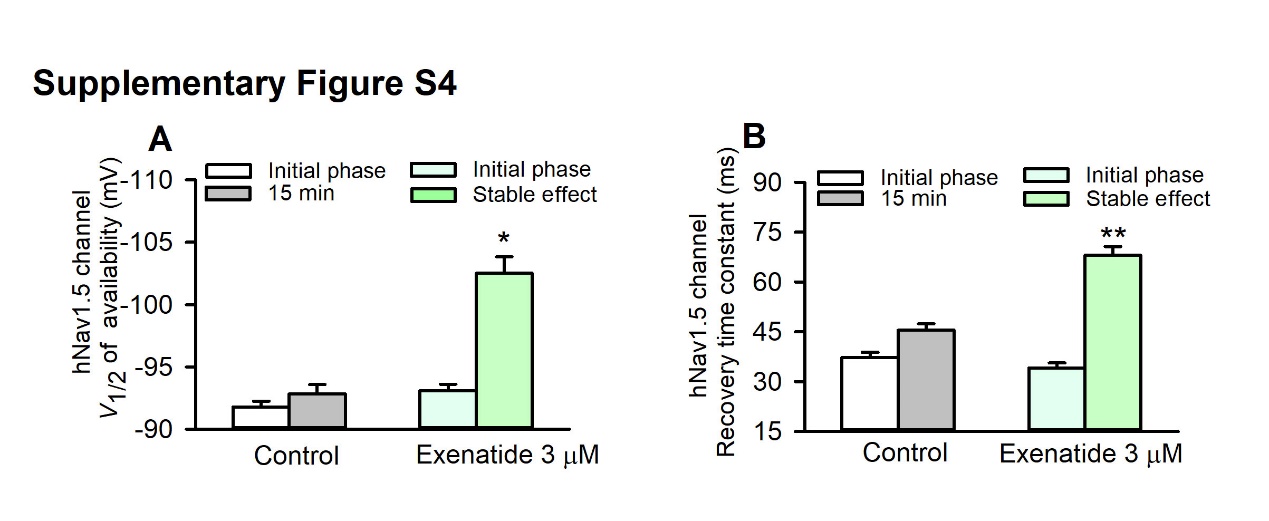
**

**Figure S4. Effects of exenatide and patch time on *V*_1/2_ of availability and recovery time constants from inactivation of *I*_Nav1.5_.** A. The *V*_1/2_ of *I*_Nav1.5_ availability with patch time (n=5; P > 0.05, non-paired Student’s *t*-test) or with 3 μM exenatide reaching a stable effect (n=6; P < 0.05, non-paired Student’s *t*-test). B. Recovery time constants from inactivation of *I*_Nav1.5_ with patch time (n=6; P > 0.05, non-paired Student’s *t*-test) or with 3 μM exenatide reaching a stable effect (n=5; P < 0.01, non-paired Student’s *t*-test).


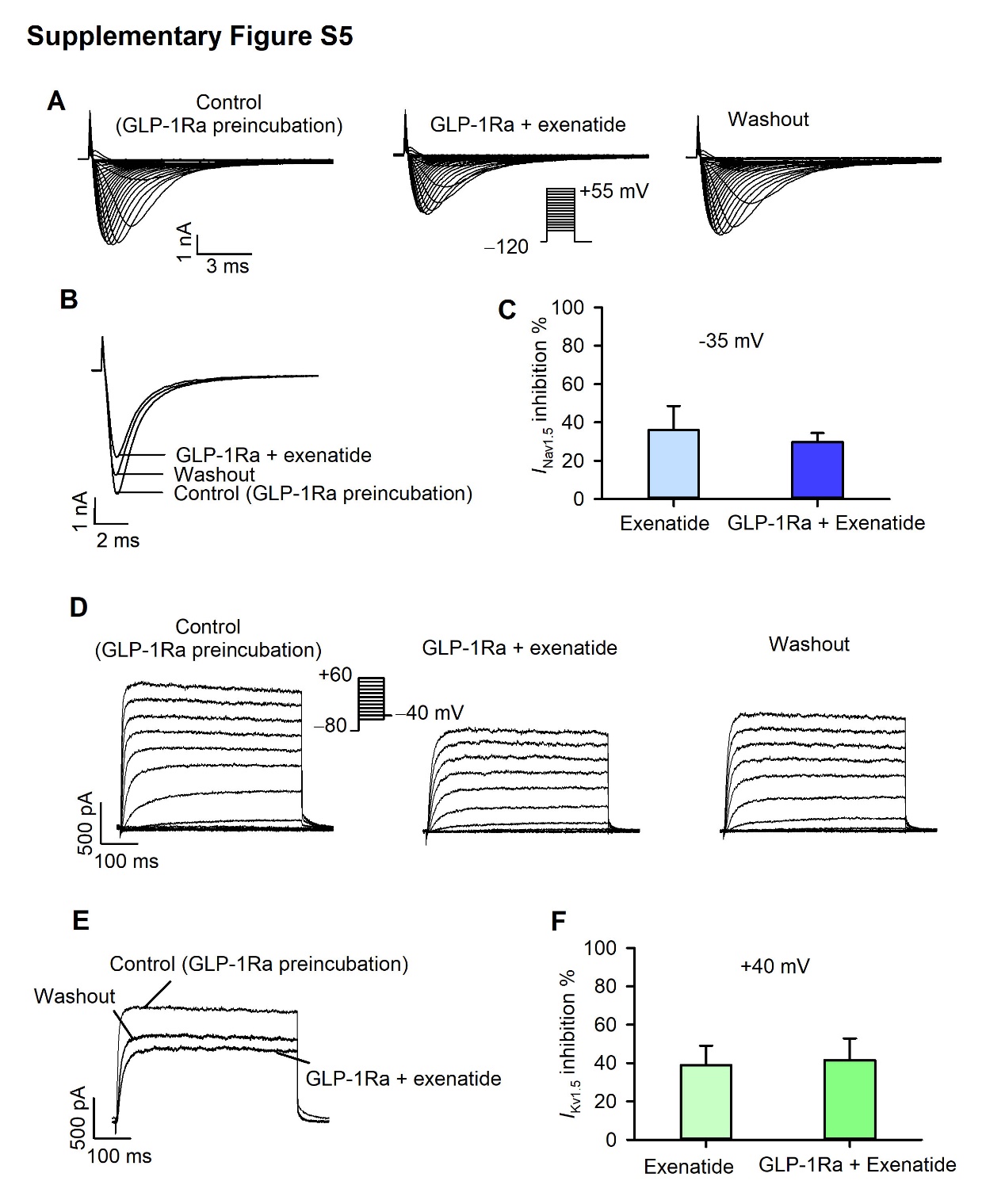


**Figure S5. Influences of GLP-1Ra on the effect of exenatide on hNav1.5 and hKv1.5 currents.** A. Representative voltage-dependent currents recorded with 100-ms voltage steps to between -100 to +55 mV from -120 mV under control (recorded after 1-h pre-incubation with 1 μM GLP-1Ra), addition of 3 μM exenatide, and after washout in HEK 293-hNav1.5 cells. B. Original current traces recorded at -35 mV indicated in (A). C. Percentage inhibition of *I*_Nav1.5_ to show the effect of exenatide in the absence and presence of GLP-1Ra at -35 mV (n=6; P > 0.05 vs. exenatide alone, non-paired Student’s *t*-test). For this experiment, a bath solution containing 1 μM GLP-1Ra was used. D. Representative voltage-dependent currents recorded with 500-ms voltage steps between -80 to +40 mV and back to -40 mV under control (recorded after 1-h pre-incubation with 1 μM GLP-1Ra), addition of 3 μM exenatide to the bath solution, and after washout in HEK 293-hKv1.5 cells. E. Original current traces recorded at +40 mV indicated in (D). F. Percentage inhibition of *I*_Kv1.5_ to show the effect of exenatide in the absence and presence of GLP-1Ra at +40 mV (n=6; P > 0.05 vs. exenatide alone, non-paired Student’s *t*-test). For this experiment, a bath solution containing 1 μM GLP-1Ra was used.
